# Association between the 30-s chair stand test and peak oxygen uptake in patients undergoing cardiopulmonary exercise testing: A large retrospective internal and external validation study

**DOI:** 10.1016/j.ijcha.2026.102018

**Published:** 2026-09-22

**Authors:** Takatoshi Iwasaki, Yoshiki Hata

**Affiliations:** a Department of Rehabilitation, Minamino Cardiovascular Hospital, Japan; b Hachioji Minamino Cardiac Rehabilitation Clinic, Japan; c Department of Cardiology, Minamino Cardiovascular Hospital, Japan

**Keywords:** 30-s chair stand test, Cardiopulmonary exercise testing, Exercise capacity, Functional assessment, Peak oxygen uptake

## Abstract

**Background:**

Peak oxygen uptake (peak VO_2_) assessed by cardiopulmonary exercise testing (CPET) is the gold standard for evaluating exercise capacity, but its routine use is limited in many clinical settings. This retrospective study investigated the association between 30-s chair stand test (CS-30) performance and peak VO_2_ and developed a prediction model with internal (Facility A) and external (Facility B) validation.

**Methods:**

This study initially included 3652 patients who underwent CPET and physical function assessments between January 2020 and December 2025. After excluding 75 patients with a respiratory exchange ratio ≤ 1.04, 3577 patients were included in the analysis. A multivariable linear regression model including CS-30 performance, age, sex, and body mass index was developed using Facility A data and externally validated using Facility B data.

**Results:**

CS-30 performance was independently associated with peak VO_2_ after multivariable adjustment. In the external validation cohort, predicted peak VO_2_ showed a moderate correlation with observed peak VO_2_ (*r* = 0.654, *p* < 0.001), with a root mean square error of 4.73 mL/kg/min and a mean bias of −2.23 mL/kg/min.

**Conclusions:**

CS-30 performance was independently associated with peak VO_2_. Although the prediction model showed moderate performance in the external validation cohort, CS-30 may serve as a simple complementary functional assessment associated with exercise capacity in clinical settings where CPET is not readily available.

## 1 Introduction

Peak oxygen uptake (peak VO_2_), measured by cardiopulmonary exercise testing (CPET), is the gold standard for assessing exercise capacity and plays a central role in evaluating disease severity and prognosis in patients with cardiovascular and chronic diseases [1], [2]. Large cohort studies and meta-analyses have consistently demonstrated that reduced peak VO_2_ is strongly associated with increased risks of all-cause mortality and cardiovascular events across a wide range of populations [3], [4].

Despite its clinical importance, CPET is a resource-intensive procedure that typically requires a specialized laboratory setting. The test involves a metabolic cart for gas exchange analysis, an ergometer (bicycle or treadmill), electrocardiographic and blood pressure monitoring, and trained personnel. In addition, time-consuming preparation, including calibration of gas sensors and flowmeters and fitting of a sealed face mask, is required. As the test also requires 10–15 min of symptom-limited or near-maximal exercise effort, it may impose physical and psychological burdens on some patients. These requirements limit the routine implementation of CPET in many clinical settings [5], [6].

There has been growing interest in simple functional assessments that may reflect exercise capacity when CPET is not readily available. Previous studies have reported associations between peak VO_2_ and physical function measures, such as the 6-min walk distance, handgrip strength, and lower limb muscle strength [7], [8], [9], [10], [11]. However, these assessments may be limited by space requirements, specialized equipment, or modest correlations with aerobic capacity [7], [8], [9], [10], [11].

The 30-s chair stand test (CS-30), originally developed as a measure of lower limb function, offers a simple and equipment-free alternative that can be performed in confined clinical environments [12]. Although recent studies have suggested an association between CS-30 performance and exercise capacity [13], [14], these investigations have been limited by their single-center designs, relatively small sample sizes, and insufficient adjustment for key confounders.

Whether a simple functional test such as CS-30 can reasonably reflect exercise capacity across heterogeneous clinical populations remains uncertain. Therefore, the present study aimed to investigate the association between CS-30 performance and peak VO_2_ using large-scale retrospective data and to develop and internally and externally validate a prediction model for peak VO_2_ based on CS-30 performance and readily available clinical characteristics.

## 2 Methods

### 2.1 Study design and setting

This two-center retrospective observational study initially included 3652 patients who underwent CPET and physical function assessments between January 2020 and December 2025. The study population included adults aged 21–91 years and represented a broad clinical population encountered in routine practice. Data were collected from two facilities. Facility A (the development cohort) is a cardiovascular hospital that primarily treats outpatients with lifestyle-related diseases. Facility B (the external validation cohort) provides care for patients participating in structured cardiac rehabilitation programs. Data from Facility A were used for model development and internal validation, whereas data from Facility B were used for independent external validation. The study protocol conforms to the ethical guidelines of the 1975 Declaration of Helsinki as reflected in a priori approval by the institution's human research committee (approval number: MJ-101), and all participants provided written informed consent.

### 2.2 Study population

Patients were excluded if a physician judged them as unable to perform CPET because of neurological disease interfering with coordination/balance, respiratory disorders, locomotor disorders (e.g., known lower extremity vascular insufficiency, severe osteoarthritis, or gait unsteadiness), or postural hypotension that could interfere with exercise testing. Patients were also excluded if data for CS-30 were missing, or if they failed to achieve sufficient effort during CPET. Maximal effort was assessed using the respiratory exchange ratio (RER). Based on guideline recommendations, an RER value around 1.05 is generally considered indicative of near-maximal effort [5]. In the present study, a pragmatic cutoff was applied, and tests with an RER ≤ 1.04 were excluded to balance data quality with real-world feasibility in clinical populations.

### 2.3 Data collection

Clinical data including age, sex, body mass index (BMI), and comorbidities (ischemic heart disease, diabetes mellitus, hypertension, and dyslipidemia) were retrospectively obtained from the patients' medical records.

### 2.4 CPET

Symptom-limited CPET was performed using a bicycle ergometer with a ramp protocol designed to achieve exhaustion within approximately 8–12 min. Oxygen uptake was measured breath-by-breath using a metabolic gas analysis system. Continuous electrocardiographic monitoring and blood pressure measurements were performed throughout the test.

### 2.5 Physical function assessment

CS-30 was performed according to a standardized protocol [10], [12]. The participants were wearing their usual footwear and were seated on a 40-cm-high, hard, uncushioned backless chair positioned against a wall for stability, with their feet shoulder-width apart and arms folded across the chest. At the “start” signal, they were instructed to repeatedly stand to the fully upright position (with complete hip and knee extension) and return to the seated position as many times as possible for 30 s. Prior to the assessment, the participants were allowed five practice trials, followed by a sufficient rest period. Subsequently, a single test attempt was performed and recorded as the absolute number of repetitions. If a participant had completed more than half of the standing motion when 30 s had elapsed, it was counted as a full repetition. In Facility A, five-times sit-to-stand test (5STS) data were also available and were used for an exploratory correlation analysis with CS-30 performance.

### 2.6 Statistical analysis

Continuous variables are presented as the mean (standard deviation [SD]) and categorical variables as counts and percentages. Correlations were evaluated using Pearson's correlation analysis. Sex was coded as a binary variable (male = 1, female = 0). Age was categorized into five groups (<50, 50–59, 60–69, 70–79, and ≥ 80 years) for descriptive analyses. Differences in peak VO_2_ and CS-30 across age groups and between sexes were evaluated using two-way analysis of variance (ANOVA).

A multivariable linear regression model was constructed in the development cohort using the forced-entry method, with age, sex, BMI, and CS-30 performance as explanatory variables. Internal validation was performed using bootstrap resampling with 2000 iterations to assess model stability and internal validity. Model performance in the external validation cohort was assessed using Pearson's correlation coefficients, coefficients of determination (R^2^), root mean square error (RMSE), mean absolute error (MAE), and Bland–Altman analysis. Model calibration was assessed by regressing observed peak VO_2_ on predicted peak VO_2_, with the regression intercept and slope reported as the calibration intercept and calibration slope, respectively. Bland–Altman analysis was used to assess agreement between predicted and observed peak VO_2_ values by calculating the mean bias (observed minus predicted) and 95% limits of agreement. Proportional bias was assessed by linear regression of the differences (observed minus predicted peak VO_2_) against the mean of the observed and predicted peak VO_2_ values. A sensitivity analysis was also performed using a more stringent peak RER cutoff of ≥1.10 to assess the robustness of the primary findings. Additional subgroup analyses were performed according to sex and major clinical characteristics (ischemic heart disease, diabetes mellitus, hypertension, and dyslipidemia) to evaluate the predictive performance of the proposed model. Statistical significance was defined as a two-sided *p*-value <0.05.

## 3 Results

Among the 3652 eligible patients, 75 with an RER ≤ 1.04 were excluded, leaving 3577 patients included in the analysis (Facility A: *n* = 2620; Facility B: *n* = 957). The mean age was 69.2 (SD 10.8) years, and 67.8% of the patients were male. The mean CS-30 performance was 21.1 (SD 5.7) repetitions, and the mean peak VO₂ was 21.8 (SD 5.7) mL/kg/min. The baseline characteristics of the study population are summarized in Table 1.

**Table 1:** Baseline characteristics of the study population stratified by facility.

Variable	Overall	Facility A	Facility B
n	3577	2620	957
Age, years, mean (SD)	69.2 (10.8)	69.1 (10.8)	69.6 (11.0)
Male sex, n (%)	2426 (67.8)	1751 (66.8)	675 (70.5)
BMI, kg/m^2^, mean (SD)	24.2 (3.8)	24.3 (3.8)	23.8 (4.0)
Ischemic heart disease, n (%)	1835 (51.3)	1169 (44.6)	666 (69.6)
Diabetes mellitus, n (%)	807 (22.6)	551 (21.0)	256 (26.8)
Hypertension, n (%)	2010 (56.2)	1441 (55.0)	569 (59.5)
Dyslipidemia, n (%)	2023 (56.6)	1357 (51.8)	666 (69.6)
CS-30, repetitions, mean (SD)	21.1 (5.7)	21.7 (5.5)	19.6 (6.0)
Peak VO_2_, mL/kg/min, mean (SD)	21.8 (5.7)	22.6 (5.7)	19.7 (5.4)

BMI, body mass index; CS-30, 30-s chair stand test; SD, standard deviation; VO_2_, oxygen uptake.

The analyses of peak VO_2_ and CS-30 were stratified by age group and sex. Peak VO_2_ decreased progressively with age and was consistently higher in males than in females across all age groups (Table 2). Similarly, CS-30 performance declined with age and was higher in males than in females across all age groups (Table 3). The differences in peak VO_2_ and CS-30 across age groups and between sexes were statistically significant (two-way ANOVA, both *p* < 0.001).

**Table 2:** Distribution of peak VO_2_ by age group and sex in the overall cohort.

Age group (years)	Male, n, mean (SD)	Female, n, mean (SD)
<50	128, 26.96 (6.54)	60, 22.14 (5.86)
50–59	405, 25.96 (6.15)	126, 21.35 (4.34)
60–69	604, 24.28 (5.49)	219, 20.58 (4.42)
70–79	919, 22.07 (4.97)	491, 18.50 (3.98)
80+	370, 19.39 (4.70)	255, 16.69 (3.47)

ANOVA, analysis of variance; SD, standard deviation; VO_2_, oxygen uptake. Differences across age groups and between sexes were statistically significant (two-way ANOVA, both *p* < 0.001).

**Table 3:** Distribution of CS-30 performance by age group and sex in the overall cohort.

Age group (years)	Male, n, mean (SD)	Female, n, mean (SD)
<50	128, 25.38 (4.96)	60, 22.03 (5.57)
50–59	405, 24.24 (5.42)	126, 21.80 (5.31)
60–69	604, 22.88 (5.48)	219, 20.72 (5.15)
70–79	919, 20.97 (5.55)	491, 19.40 (4.92)
80+	370, 18.43 (5.30)	255, 17.52 (4.92)

ANOVA, analysis of variance; CS-30, 30-s chair stand test; SD, standard deviation. Differences across age groups and between sexes were statistically significant (two-way ANOVA, both *p* < 0.001).

In the development cohort (Facility A: *n* = 2620), CS-30 performance showed a moderate positive correlation with peak VO_2_ (*r* = 0.589, *p* < 0.001; Fig. 1), indicating that higher CS-30 performance was associated with higher peak VO_2_. A multivariable linear regression model including age, sex, BMI, and CS-30 performance explained a moderate proportion of the variance in peak VO_2_ (R^2^ = 0.522, adjusted R^2^ = 0.521, *p* < 0.001; Table 4). The derived prediction equation was as follows: predicted peak VO_2_ (mL/kg/min) = 31.565–0.174 × age + 3.550 × sex −0.371 × BMI + 0.448 × CS-30. Among the predictors, CS-30 performance was independently associated with peak VO_2_ and showed the highest standardized coefficient value (β = 0.434, *p* < 0.001). Internal validation using bootstrap resampling (2000 iterations) demonstrated stable coefficient estimates with minimal optimism, indicating good internal validity. In an additional exploratory analysis, CS-30 performance showed a strong negative correlation with 5STS (*r* = −0.830, *p* < 0.001; *n* = 2517; Supplementary Table S1).

**Fig. 1:**
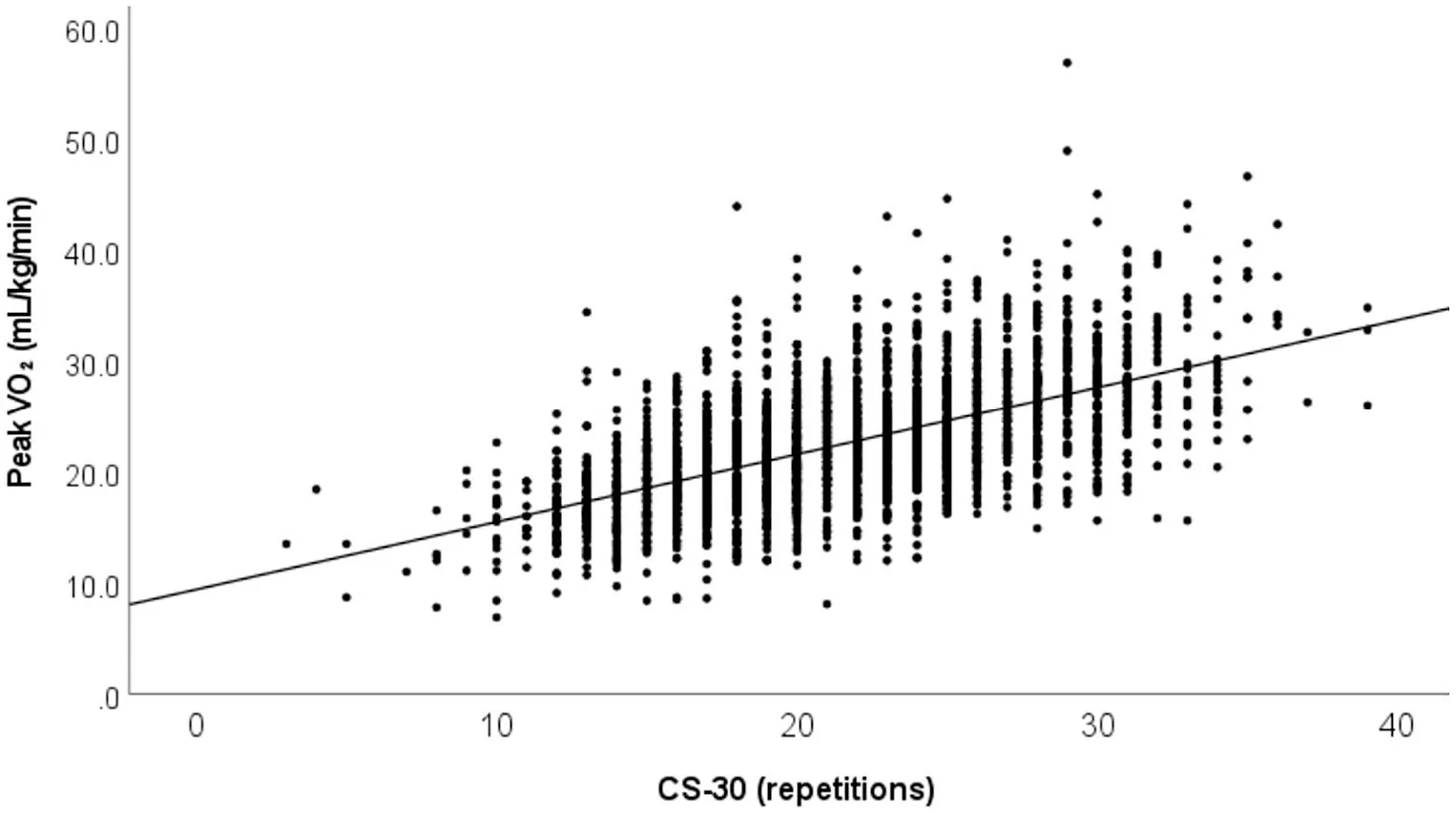
Scatter plot showing the relationship between CS-30 performance and peak VO₂ in the development cohort (Facility A: *n* = 2620). The solid line represents the linear regression line. CS-30, 30-s chair stand test; VO_2_, oxygen uptake.

**Table 4:** Multivariable linear regression model for estimating peak VO_2_ in the development cohort (Facility A).

Variable	B	SE	β	*p*-value	95% CI
Age	−0.174	0.008	−0.332	<0.001	−0.190 to −0.159
Male sex	3.550	0.169	0.295	<0.001	3.219 to 3.881
BMI	−0.371	0.022	−0.248	<0.001	−0.414 to −0.329
CS-30	0.448	0.015	0.434	<0.001	0.418 to 0.477

Values are presented as regression coefficients (B), SE, standardized coefficients (β), and 95% CI. Sex was coded as 1 for male and 0 for female. BMI, body mass index; CI, confidence interval; CS-30, 30-s chair stand test; SE, standard error; VO_2_, oxygen uptake.

In the external validation cohort (Facility B: *n* = 957), the predicted peak VO_2_ showed a moderate positive correlation with the observed peak VO_2_ (*r* = 0.654, *p* < 0.001; Fig. 2). The model demonstrated an R^2^ of 0.428, an RMSE of 4.73 mL/kg/min, an MAE of 3.78 mL/kg/min, and a mean bias of −2.23 mL/kg/min between the observed and predicted peak VO_2_ values (Table 5). The calibration intercept was 1.702 (95% CI, 0.357–3.048), and the calibration slope was 0.820 (95% CI, 0.760–0.881). Bland–Altman analysis showed a mean bias of −2.23 mL/kg/min, with 95% limits of agreement from −10.41 to 5.94 mL/kg/min (Fig. 3). A significant proportional bias was observed, with the difference between observed and predicted peak VO_2_ increasing as their mean increased (regression coefficient = 0.272, 95% CI 0.215–0.329, *p* < 0.001).

**Fig. 2:**
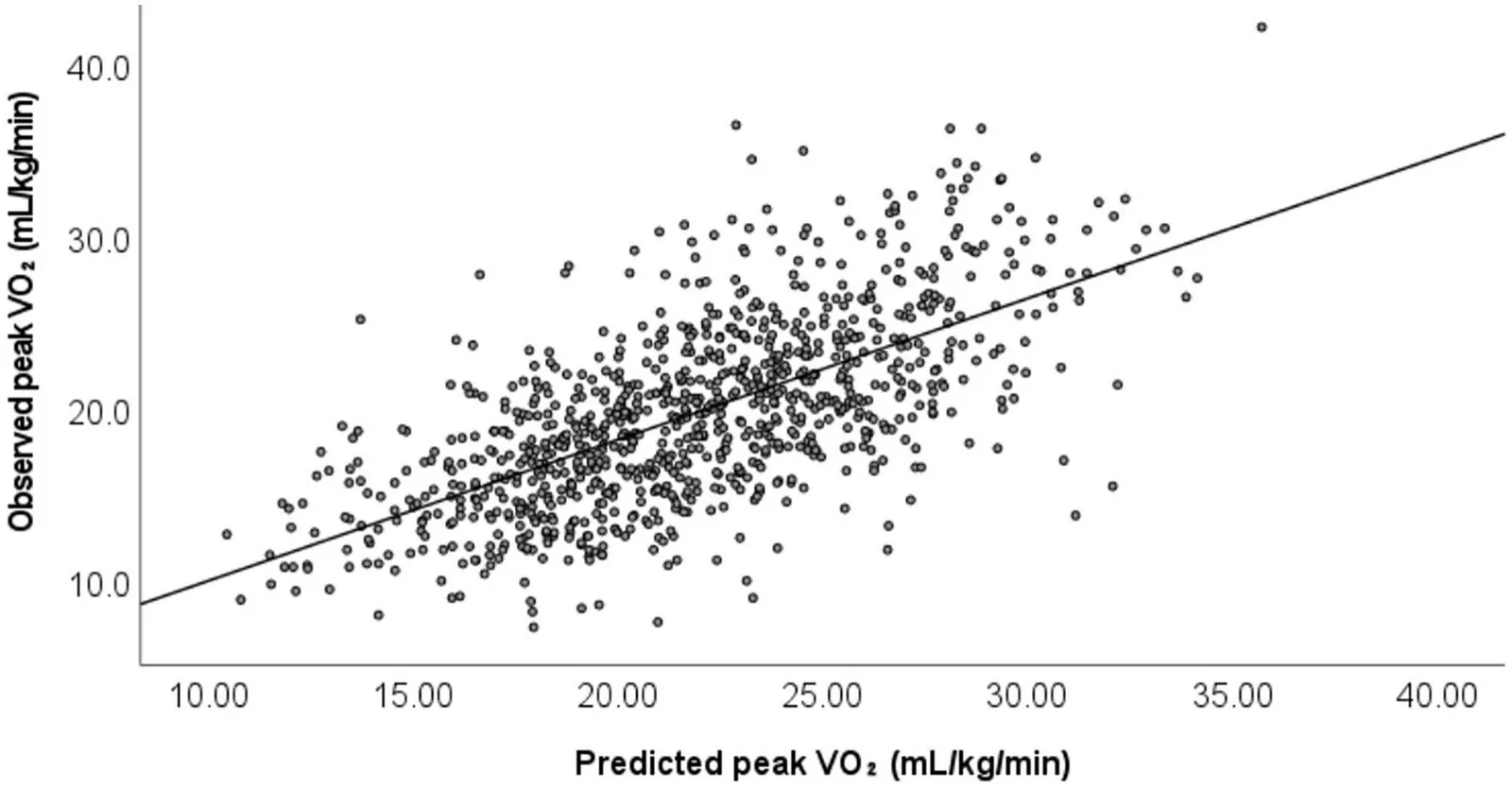
Scatter plot showing the relationship between the predicted and observed peak VO_2_ in the external validation cohort (Facility B: *n* = 957). The solid line represents the linear regression line. The model demonstrated a moderate correlation between the predicted and measured values (*r* = 0.654, *p* < 0.001; RMSE = 4.73 mL/kg/min). RMSE, root mean square error; VO_2_, oxygen uptake.

**Table 5:** Model performance for predicting peak VO_2_ in the external validation cohort (Facility B).

Metric	Value
Pearson's correlation coefficient (r)	0.654
Coefficient of determination (R^2^)	0.428
Calibration intercept	1.702 (95% CI, 0.357–3.048)
Calibration slope	0.820 (95% CI, 0.760–0.881)
RMSE (mL/kg/min)	4.73
MAE (mL/kg/min)	3.78
Mean bias (mL/kg/min)	−2.23
95% limits of agreement (mL/kg/min)	−10.41 to 5.94

CI, confidence interval; MAE, mean absolute error; bias, mean difference calculated as observed minus predicted peak VO_2_; limits of agreement, mean bias ±1.96 SD; RMSE, root mean square error; SD, standard deviation; VO_2_, oxygen uptake.

**Fig. 3:**
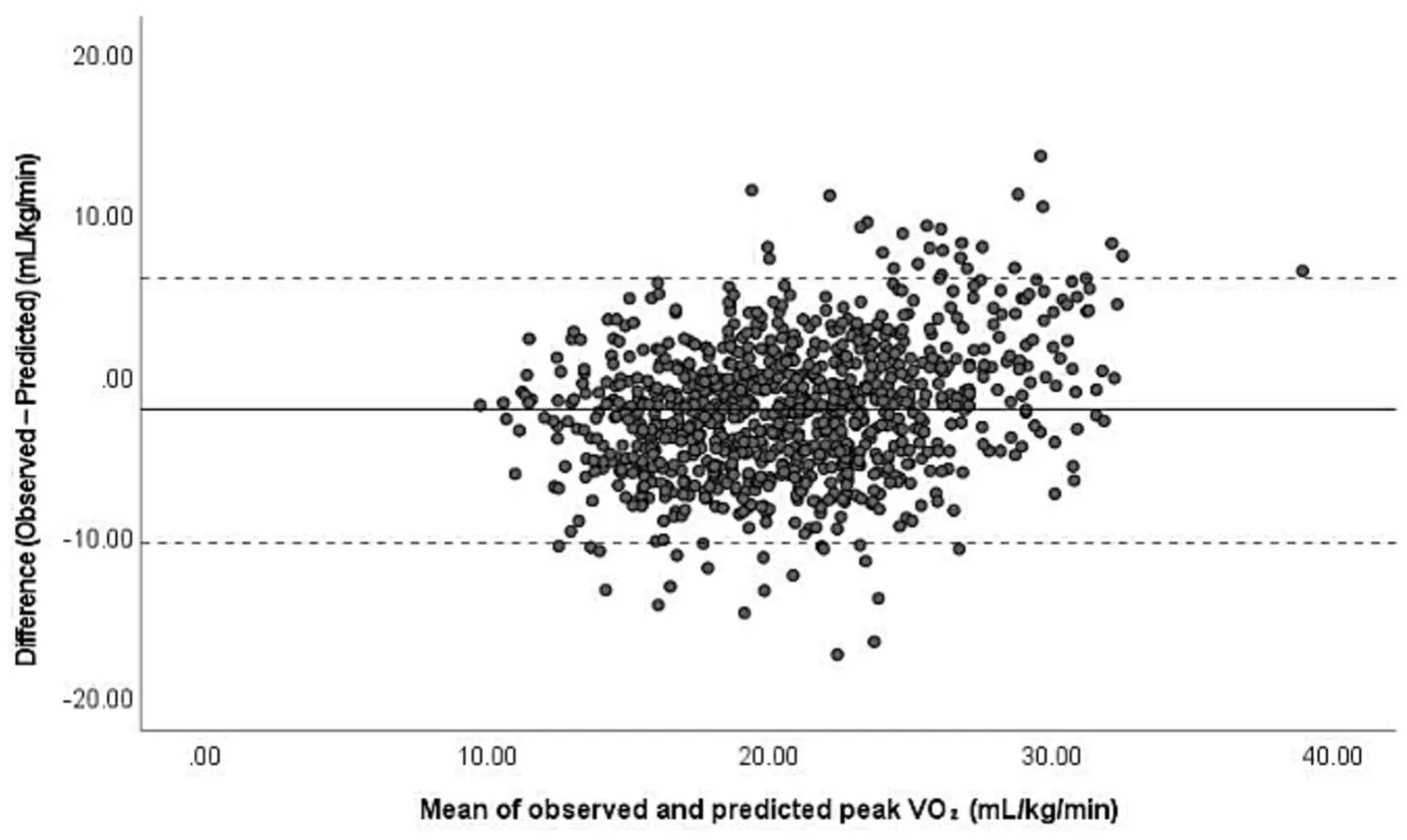
Bland–Altman plot showing agreement between observed and predicted peak VO_2_ in the external validation cohort (Facility B: *n* = 957). The mean bias (observed minus predicted peak VO_2_) was −2.23 mL/kg/min, with 95% limits of agreement from −10.41 to 5.94 mL/kg/min. The solid line represents the mean bias, and the dashed lines represent the 95% limits of agreement. VO_2_, oxygen uptake.

Additional subgroup analyses according to sex and major clinical characteristics (IHD, DM, HT, and dyslipidemia) demonstrated moderate positive correlations between CS-30 performance and peak VO_2_ across all clinical subgroups (Supplementary Table S2). In the external validation cohort, the model showed moderate correlations between predicted and observed peak VO_2_ across all examined subgroups (*r* = 0.598–0.694), although prediction errors varied modestly among subgroups (Supplementary Table S3).

In a sensitivity analysis restricted to participants with a peak RER ≥1.10, the model showed similar performance to the primary analysis, with an R^2^ of 0.511 in the development cohort and an r of 0.657, RMSE of 4.85 mL/kg/min, MAE of 3.90 mL/kg/min, and mean bias of −2.52 mL/kg/min in the external validation cohort (Supplementary Table S4).

## 4 Discussion

This large retrospective two-center study demonstrated that CS-30 performance was independently associated with peak VO₂ measured by CPET after adjustment for age, sex, and BMI. Among the variables included in the model, CS-30 showed the largest standardized coefficient. The prediction model demonstrated moderate performance in the external validation cohort, while internal validation supported the stability of the model estimates.

As CS-30 involves repeated sit-to-stand movements using large lower extremity muscle groups, it may reflect integrated lower extremity strength [15], as well as endurance, balance, neuromuscular control, and overall functional performance, all of which contribute to exercise capacity. Peak oxygen uptake reflects the integrated capacity of the cardiovascular, respiratory, and skeletal muscle systems to deliver and utilize oxygen during exercise. In particular, peripheral skeletal muscle function has been recognized as an important determinant of exercise tolerance, especially in patients with cardiovascular disease [16]. Therefore, the observed association between CS-30 performance and peak VO_2_ is biologically plausible because both measures are influenced, at least in part, by lower extremity skeletal muscle function.

Previous studies have suggested that sit-to-stand performance is influenced by lower-extremity muscle strength as well as other components of physical function [15], [17]. Taken together, these findings suggest that CS-30 reflects broader aspects of physical function rather than isolated muscle strength alone. Previous studies have also demonstrated associations between CS-30 performance and sarcopenia and other measures of functional fitness [18], [19]. Accordingly, CS-30 performance is influenced not only by aerobic exercise capacity but also by musculoskeletal and neuromuscular factors, which may partly explain the moderate association observed with peak VO_2_ in the present study.

In addition, CS-30 showed a strong correlation with the five-times sit-to-stand test (5STS), another commonly used repeated sit-to-stand assessment of lower-extremity function (Supplementary Table S1), further supporting that CS-30 reflects lower-extremity functional performance.

Associations between CS-30 performance and exercise tolerance have also been reported in specific clinical populations, including older adults with cardiovascular disease and patients with cancer [13], [14]. These findings further support the potential clinical utility of CS-30 as a simple functional assessment. Taken together, the present findings and previous studies suggest that CS-30 may provide clinically meaningful information regarding overall physical function and exercise capacity in routine practice.

It is essential to emphasize that CS-30 should not be interpreted as a direct surrogate for CPET-derived peak VO_2_. CPET remains the gold standard for detailed physiological assessment of exercise capacity [5], [6], [20]. In the present study, although the prediction model showed a moderate correlation with observed peak VO_2_ in the external validation cohort, Bland–Altman analysis demonstrated a mean bias of −2.23 mL/kg/min with relatively wide 95% limits of agreement (−10.41 to 5.94 mL/kg/min), together with a significant proportional bias. The calibration intercept of 1.702 and calibration slope of 0.820 further indicated deviation from perfect calibration in the external validation cohort. These findings indicate that prediction errors were not uniform across the range of peak VO_2_ values and limit the use of the proposed equation for precise individual-level estimation.

Nevertheless, the comparable model performance observed in the sensitivity analysis using a more stringent RER cutoff supports the robustness of the overall findings. Given that CS-30 is simple, inexpensive, and feasible in routine outpatient settings, it may serve as a simple complementary functional assessment associated with exercise capacity in clinical situations where formal CPET is unavailable or difficult to perform.

### 4.1 Limitations

This study had several limitations. First, the retrospective observational design precluded causal inference. Second, data on pharmacological treatments, habitual physical activity, and detailed measures of cardiac function, including echocardiographic parameters, were not systematically available. Therefore, the potential influence of these factors on the observed associations could not be fully assessed. Third, variations in CPET protocols among the participants may have influenced peak VO_2_ measurements. Fourth, a habitual cyclist may perform better on the cycle ergometer than a non-cyclist of the same cardiovascular fitness. However, individual familiarity with cycling was not assessed, and we did not specifically adjust for cycling habits in the primary analysis. Therefore, individual familiarity with cycling may have influenced the measured peak VO_2_ values. Fifth, the moderate predictive performance, relatively wide limits of agreement, and significant proportional bias limit the use of the proposed equation for precise estimation of peak VO_2_ at the individual level. Finally, unmeasured musculoskeletal or neurological conditions may have influenced CS-30 performance independently of cardiopulmonary function.

## 5 Conclusion

The findings of this large-scale retrospective study with internal and external validation showed that CS-30 performance was independently associated with peak VO_2_. Given its simplicity and feasibility, CS-30 may serve as a simple complementary functional assessment associated with exercise capacity in clinical settings where CPET is not readily available.

## Declaration of generative AI and AI-assisted technologies in the manuscript preparation process

During the preparation of this manuscript, the authors used DeepL and ChatGPT to verify their written English. After using these tools, the authors reviewed and edited the content as needed and take full responsibility for the content of the published article.

## CRediT authorship contribution statement

**Takatoshi Iwasaki:** Writing – review & editing, Writing – original draft, Visualization, Methodology, Investigation, Formal analysis, Data curation, Conceptualization. **Yoshiki Hata:** Writing – review & editing, Supervision, Conceptualization.

## Funding

This research did not receive any specific grant from funding agencies in the public, commercial, or not-for-profit sectors.

## Declaration of competing interest

The authors declare that they have no known competing financial interests or personal relationships that could have appeared to influence the work reported in this paper.

## Appendix A Supplementary data

Supplementary Table S1. Correlation between the 30-second chair stand test (CS-30) and the five-times sit-to-stand test (5STS) in the development cohort (Facility A).
