# Supplementary material for: Association between the 30-s chair stand test and peak oxygen uptake in patients undergoing cardiopulmonary exercise testing: A large retrospective internal and external validation study
Source: Int J Cardiol Heart Vasc. 2026 Sep 22;66:102018. doi: 10.1016/j.ijcha.2026.102018 (PMC13633863; doi:10.1016/j.ijcha.2026.102018)
Supplement: Supplementary file 1 [file mmc1.docx]

**Supplementary Table S1.** Correlation between the 30-second chair stand test (CS-30) and the five-times sit-to-stand test (5STS) in the development cohort (Facility A).

| **Variable** | **Pearson's r** | **P value** | **N** |
| --- | --- | --- | --- |
| CS-30 vs. 5STS | -0.830 | <0.001 | 2517 |

Pearson correlation analysis. Analysis was performed using participants with available data in the development cohort (Facility A).

**Supplementary Table S2.** Correlation between CS-30 performance and peak oxygen uptake according to clinical subgroups in the development cohort (Facility A).

| **Clinical subgroup** | **n** | **Pearson's r** | **P value** |
| --- | --- | --- | --- |
| Overall | 2620 | 0.589 | <0.001 |
| Sex |  |  |  |
| Female | 869 | 0.545 | <0.001 |
| Male | 1751 | 0.582 | <0.001 |
| **Ischemic heart disease** |  |  |  |
| No | 1451 | 0.561 | <0.001 |
| Yes | 1169 | 0.614 | <0.001 |
| **Diabetes mellitus** |  |  |  |
| No | 2069 | 0.588 | <0.001 |
| Yes | 551 | 0.582 | <0.001 |
| **Hypertension** |  |  |  |
| No | 1179 | 0.595 | <0.001 |
| Yes | 1441 | 0.583 | <0.001 |
| **Dyslipidemia** |  |  |  |
| No | 1263 | 0.568 | <0.001 |
| Yes | 1357 | 0.612 | <0.001 |

Pearson's correlation coefficients represent the association between CS-30 performance and peak VO_2_ within each clinical subgroup.

**Supplementary Table S3.** Predictive performance of the proposed model according to clinical subgroups in the external validation cohort (Facility B).

| **Subgroup** | **n** | **Pearson’s r** | **Bias** | **MAE** | **RMSE** |
| --- | --- | --- | --- | --- | --- |
| Overall | 957 | 0.654 | −2.23 | 3.78 | 4.73 |
| **Sex** |  |  |  |  |  |
| Female | 282 | 0.598 | −1.68 | 3.19 | 3.93 |
| Male | 675 | 0.602 | −2.47 | 4.03 | 5.03 |
| **Ischemic heart disease** | | |  |  |  |
| No | 291 | 0.633 | −3.55 | 4.28 | 5.32 |
| Yes | 666 | 0.643 | −1.66 | 3.57 | 4.45 |
| **Diabetes mellitus** | | |  |  |  |
| No | 701 | 0.666 | −2.11 | 3.74 | 4.69 |
| Yes | 256 | 0.604 | −2.58 | 3.91 | 4.83 |
| **Hypertension** | | |  |  |  |
| No | 388 | 0.694 | −2.18 | 3.85 | 4.73 |
| Yes | 569 | 0.614 | −2.27 | 3.74 | 4.73 |
| **Dyslipidemia** | |  |  |  |  |
| No | 291 | 0.671 | −2.92 | 3.98 | 5.01 |
| Yes | 666 | 0.650 | −1.93 | 3.70 | 4.60 |

Bias was calculated as observed minus predicted peak VO_2_. MAE, mean absolute error; RMSE, root mean square error; VO_2_, oxygen uptake.

**Supplementary Table S4.** Sensitivity analysis using a more stringent peak RER cutoff (≥1.10).

| **Model performance** | **Primary analysis** | **Sensitivity analysis** |
| --- | --- | --- |
| Development cohort, n | 2,620 | 2,336 |
| R^2^ | 0.522 | 0.511 |
| External validation cohort, n | 957 | 874 |
| Pearson’s r | 0.654 | 0.657 |
| RMSE, mL/kg/min | 4.73 | 4.85 |
| MAE, mL/kg/min | 3.78 | 3.90 |
| Mean bias, mL/kg/min | −2.23 | −2.52 |

The primary analysis included participants with peak RER ≥1.05, whereas the sensitivity analysis was restricted to those with peak RER ≥1.10. Mean bias was calculated as observed minus predicted peak VO_2_.

MAE, mean absolute error; R^2^, coefficient of determination; RER, respiratory exchange ratio; RMSE, root mean square error; VO_2_, oxygen uptake.
